# Non-traumatic keratitis due to *Colletotrichum truncatum*

**DOI:** 10.1099/jmmcr.0.005047

**Published:** 2016-08-30

**Authors:** Reina Llamos, Abdullah M. S. Al-Hatmi, Gerardo Martínez, Ferry Hagen, Rosario Velar, Alexeide de la Caridad Castillo Pérez, Jacques F. Meis, María T. Illnait-Zaragozí

**Affiliations:** ^1^​ Department of Microbiology, Ophthalmological Institute “Ramón Pando Ferrer”, Havana, Cuba; ^2^​ Fungal Biodiversity Centre (CBS-KNAW), Utrecht, the Netherlands; ^3^​ Department of Bacteriology and Mycology, Tropical Medicine Institute “Pedro Kouri”, Havana, Cuba; ^4^​ Department of Medical Microbiology and Infectious Diseases, Canisius-Wilhelmina Hospital, Nijmegen, the Netherlands; ^5^​ Clinical Department, Ophthalmological Hospital “Ramón Pando Ferrer”, Havana, Cuba; ^6^​ Department of Medical Microbiology, Radboud University Medical Center, Nijmegen, the Netherlands

**Keywords:** keratomycosis, mycotic keratitis, *Colletotrichum*

## Abstract

**Introduction:**

The fungal genus *Colletotrichum* is an uncommon cause of human infections. It has been implicated in cutaneous phaeohyphomycosis, artritis and keratitis secondary to traumatic implantation.

**Case presentation:**

We report two cases of keratitis due *Colletotrichum truncatum* in middle-aged, immunocompetent persons without history of trauma. The aetiological agents were identified based on DNA sequencing. Azoles and echinocandins showed high minimal inhibitory concentrations while amphotericin B was ≤ 0.25  mg l^−1^. Both patients failed topical antifungal treatment and needed penetrating keratoplasty with a favourable outcome.

**Conclusion:**

*C. truncatum* caused keratomycosis which did not respond to topical antifungal agents. To the best of our knowledge these are the first reported cases of keratitis due to this fungus in Cuba and Latin-America and highlights the expanding spectrum of fungal agents causing eye infections.

## Introduction

Mycotic keratitis or keratomycosis is a general term for fungal infection of the cornea. It is a major cause of unilateral (and sometimes bilateral) blindness worldwide with a high prevalence in tropical and subtropical regions, particularly in rural low-resource settings (Gopinathan *et al*., 2002; Thomas & Kaliamurthy, 2013). It can be caused by a wide variety of fungi. *Candida *is considered the most common one followed by *Aspergillus*. There are several reported cases due to other fungal agents and it has been shown that any saprophytic fungus may cause an exogenous infection of the eye (Kalkanci & Ozdek, 2011). Species of the genus *Colletotrichum*, better known as plant pathogens, are considered an unusual but emerging cause of human infections. This fungus has been described as aetiological agent of cutaneous phaeohyphomycosis, artritis and keratitis after traumatic implantation (Chakrabarti *et al.*, 2008; Shivaprakash *et al*., 2011; Figtree *et al.*, 2013; Allton *et al.*, 2015; Cho *et al.*, 2015; Squissato *et al*., 2015). In Latin-America, *Colletotrichum truncatum* has never been reported as cause of human infection. Here we report two cases in Cuba of keratitis caused by this fungus occurring in immunocompetent persons without any history of trauma.

## Case Report

### Case 1

A 41-year-old, otherwise healthy male patient, pig farmer by profession from Ciego de Avila was admitted to a local hospital on 21 February 2010 because of burning ocular pain, blurred vision and redness of the left eye. He could not recall any ocular trauma. Empirical topical antibacterial treatment with ceftazidim (3 %) and vancomycin (3 %) was instituted (one eye drop every hour). The reaction to therapy was poor with increasing infiltration and ulcer extension. Corneal scraping grew methicillin-resistant *Staphylococcus aureus *(susceptible to amikacin, ciprofloxacin and gentamicin) and treatment was changed to amikacin (3 %) and ceftriaxone (3 %) eye drops every 30 min. The first few days the patient experienced a slight improvement but after a week clinical manifestations worsened, so he was referred to the Ophthalmological Institute 'Ramón Pando Ferrer' in Havana. At arrival the patient had slight oedema of the upper and lower lids of his left eye, scanty yellow secretions, intense cilium-conjunctival injection and a dense corneal yellowish infiltrate leaving only 1 mm of the peripheral limbo clear. Slit lamp examination showed central extensive epithelial defects with irregular and thick borders involving all corneal layers. There were no satellite lesions or evidence. No defects were observed in the anterior segment and his right eye was normal. Ocular ultrasound examination on 10 March 2010 showed a vitreous cavity without opacities and normal retina. Ten days later a filamentous fungus from corneal scrapings was reported and the patient started treatment with miconazole (1 %) one eye drop every hour, ketoconazole (1 %) one eye drop three times per day, and oral ketoconazole (200 mg daily for 7 days). On 22 March, he underwent penetrating keratoplasty and miconazole was replaced by natamycin (5 %) eye drops every 30 min. Eight days later topical ketoconazole was stopped and empirical oral moxifloxacin (400 mg daily for 7 days) was added. Natamycine was continued until discharge on 7 April 2010. The outcome was favourable with a five-year follow up.

### Case 2

A 70-year-old male patient from Havana with a history of asthma was admitted to the emergency service at a local polyclinic on 8 July 2010 because loss of vision, discomfort and secretions in his right eye. He denied any ocular trauma, and empirical treatment with gentamicin (3 %) eye drops every 4 h and cold compresses were initiated. Two days later he went to the Ophthalmological Institute 'Ramón Pando Ferrer' because of worsening of the ocular symptoms. At that moment physical examination showed abundant secretions, eyelid oedema, cilium-conjunctival injection, satellite injuries and hypopyon; the slit lamp examination showed a corneal defect (6 mm) with a central diffuse infiltrate (5 mm) and hypopyon (1.5 mm). A corneal scraping was taken and the treatment was changed to ceftazidim (5 %) and vancomycin (3 %) eye drops every 30 min. Five days later growth of a filamentous fungus from corneal scrapings was reported triggering change of local treatment to miconazole (1 %), natamycin (5 %) and moxifloxacin (5 %) (one eye drop every hour for 7 days). No improvement was observed after 48 h of antifungal treatment and the ocular ultrasound showed a fixed retina and no vitreous or choroid abnormalities. On 19 July the patient received a penetrating keratoplasty due to persistent blepharospasms, eyelid oedema and an intense cilium-conjunctival injection with risk of perforation. This change in treatment was successful and he was discharged 48 h later. He continued topical natamycin for another two weeks. The outcome was favourable with a five-year follow up.

## Investigations

Corneal scrapings from the ulcers were taken by an experienced ophthalmologist under aseptic precautions. The samples were immediately sent to the microbiology laboratory. Unfortunately, direct microscopic examination with 10 % KOH wet mount and Gram staining was not possible because the paucity of samples. Each corneal scraping was inoculated on blood agar, chocolate agar, brain-heart infusion agar (BHIA) and Sabouraud's dextrose agar (SDA). Blood agar, chocolate agar and BHIA were incubated at 37 °C while SDA was incubated at 25 °C. Cultures were checked daily and were considered positive when growth of the same organism was demonstrated on two or more solid media and/or if there was confluent growth at the site of inoculation on at least one solid medium. SDA became positive for a filamentous fungus after five days. No other bacteria or fungi were recovered. Colonies were flat, whitish with beige periphery, grey speckles and buff reverse. The cultures were found to be non-sporulating when examined microscopically and were sent to the National Mycology Reference Center in Nijmegen, the Netherlands, and to the CBS-KNAW Fungal Biodiversity Centre in Utrecht, the Netherlands.

**Fig. 1.**
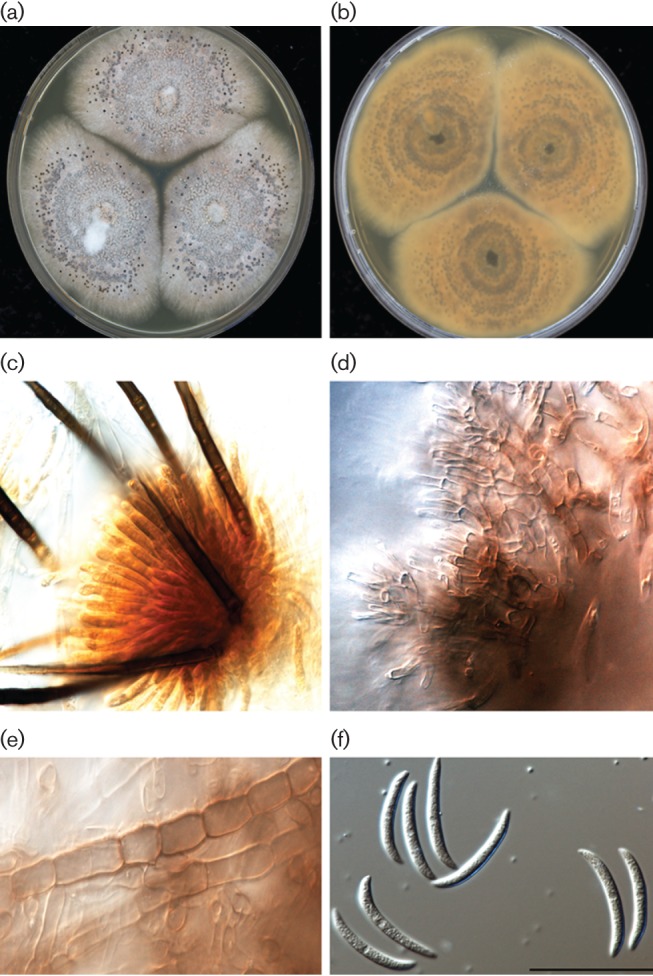
Morphological description of *Colletotrichum truncatum*. (a) Growth of the isolate on MEA, slightly olivaceous-grey to iron-grey; (b) brown to pale reverse pigmentations; (c) setae; (d) conidiogenous cells; (e) septate hyphae and conidiophores; (f) conidia. Pictures were taken using a Nikon digital-sight DS-5 M camera attached to the microscope. Bars, 10 µm

Preliminary identification to genus level was carried out according to the macro- and micro-morphology on 2 % malt extract agar (MEA; Oxoid) incubated at 24 °C. Colonies grew at a medium speed; after incubation for one week they were flat, with no aerial mycelium, buff-coloured and covered with olivaceous to iron-grey grains and reverse side brownish to pale olivacious-grey (Fig. 1a and 1b). Slide cultures were examined with a light microscope (Eclipse 80i; Nikon) and showed setae and conidiophores formed directly on hyphae with the following morphologies: setae 80–150 µm long with cylindrical base 4–6 µm diameter and 2–3 septate (Fig. 1c); conidiophores septate, branched and densely clustered (Fig. 1d); septated cylindrical forms (Fig. 1e); and conidia aseptate, smooth-walled, central part of slightly curved with round and truncate base (15–20 × 3–4 µm) (Fig. 1f). All the structures were hyaline to pale brown. Both isolates were identified by internal transcribed spacer (ITS2) sequencing and the molecular identification was reported as *Colletotrichum *species. Further molecular testing, based on DNA sequencing using a combination of different primer sets and the MycoBank (CBS-KNAW) reference database indicated that the fungal identification was most consistent with *Colletotrichum truncatum*. The internal transcribed spacer (ITS), D1/D2 region, and beta-tubulin locus were sequenced and a blast search identified both isolates as *Colletotrichum truncatum* (Genbank accession numbers KX355742 - KX355747). Some entries indicated high homology with sibling species, including *Colletotrichum capsici* and *Colletotrichum dematium.* *In vitro* antifungal susceptibility testing of amphotericin B, fluconazole, itraconazole, voriconazole, posaconazole, isavuconazole, anidulafungin and micafungin [Clinical and Laboratory Standards Institute (CLSI), 2008; Al-Hatmi *et al.,* 2015] showed high minimal inhibitory concentrations (MIC) for all drugs except amphotericin B (Table 1).

Subcultures were deposited in the reference collection of the CBS-KNAW Fungal Biodiversity Centre, Utrecht, the Netherlands under accession numbers CBS 134303 and CBS 134304.

## Discussion

Worldwide reports consistently list keratitis with corneal ulceration as the second major cause of blindness. The frequency and spectrum of the aetiological agents varies from place to place as several factors like climatic, geographical and socio-economic conditions may play a role in modulating its incidence and prevalence (Kalkanci & Ozdek, 2011; Shah *et al.,* 2011; Ansari *et al.,* 2013). For example, in central China and the USA keratitis is rarely reported (0.015 % and 1–1.2 %, respectively) (Gorscak *et al.,* 2007; Cao *et al.,* 2014), while in India, the regional distribution of corneal ulcers rank between 7.3 % in the north to 40 % in the south (Chowdhary & Singh, 2005; Thomas & Kaliamurthy, 2013). Cuba is an archipelago of islands located in the Caribbean Sea with tropical wet weather and socio-economic conditions (mainly agricultural) which could favour the occurrence of fungal keratitis. However, there are no published data on keratomycosis in Cuba.

Most studies done on mycotic keratitis have listed trauma, especially with plant material during farming, as the most common risk factor (44 to 55 % of the patients). In developed countries farming has turned more industrialized, however the widespread use of contact lenses has become one of the most frequent causes of keratomycosis. Other risk factors include corneal abrasion, foreign body implantation, use of topical corticosteroids and systemic diseases such as diabetes mellitus (Thomas, 2003). Surgical trauma may also predispose to this infection especially in elderly patients (Sharma, 2002). Most keratomycosis cases due to *Colletotrichum* species report a history of ocular trauma. Surprisingly, none of the two Cuban patients recalled any ocular trauma. Since traumatic implantation seems to be important for the initiation of *Colletotrichum* infections our patients probably suffered some minor unnoted injury (as was confirmed by slit lamp examination in both cases). Prior or concomitant bacterial or viral infections and antibiotic treatment could also act as predisposing factor. Because both patients resided more than 450 km apart and clinical manifestations in case 2 started five months later compared with case 1, the possibility of cross-infection with the same strain or an outbreak is highly unlikely.

Early diagnosis and treatment are important in preventing complications. Ophthalmologists have long maintained that it is possible to distinguish fungal from bacterial lesions based on clinical signs. Fungal keratitis has been considered a suppurative corneal ulcer with spiculated borders, satellite lesions, hypopyon, or posterior chamber endophthalmitis that fails to respond to antibacterial treatment (Ansari *et al.,* 2013). In a large series from Ghana and India, cases were systematically examined for specific features. Serrated infiltrate margins and raised surface profiles were independently associated with fungal keratitis, while anterior chamber fibrin was independently associated with bacterial keratitis (Thomas *et al*., 2005). However, accurate identification of the fungal pathogen at the species level is crucial for clinical outcome since some causative agents are refractory to antifungal treatment (Thomas & Kaliamurthy, 2013).

In accordance with Shiraishi *et al. *(2011), in our patients the site of infection was also located in the anterior corneal stroma and the hypopyon disappeared a few days after the initiation of antifungal therapy suggesting it was most likely an inflammatory response to the infection. This may be related to the temperature sensitivity of *Colletotrichum*.

Previous studies suggested that surgical debridement is required for cases with a poor response to antifungal therapy (Shiraishi *et al.,* 2011). About a week after starting with antifungal treatment, both Cuban patients received a penetrating keratoplasty because of lack of response to medical treatment. No evidence of recurrence was observed after a five-year follow-up period.

A large variety of species of *Fusarium* (Al-Hatmi *et al*., 2016a) is the most common cause of keratitis and is initiated by inoculation of the fungus during trauma (Klotz *et al.,* 2000; Kalkanci, & Ozdek, 2011). Other saprophytic fungi including *Aspergillus*,* Acremonium*,* Exophiala*,* Helminthosporium*,* Paecilomyces*,* Penicillium*,* Phialophora*,* Scedosporium*,* Neoscytalidium*,* Scopulariopsis *and *Trichophyton *spp., and *Glomeromycetes* (previously *Zygomycetes*) have also been reported (Kalkanci & Ozdek, 2011; Sharma *et al.,* 2014). In addition dematiaceous fungi have become an important aetiological agent in the last centuries (Garg *et al.,* 2000). The genus *Colletotrichum* comprises several hundred species with a macroscopic appearance (colony color and texture) clearly different from species of *Aspergillus *and *Fusarium* (Midha *et al.,* 1996; Guarro *et al.,* 1999). In humans,* Colletotrichum coccodes*,* Colletotrichum crassipes* and *Colletotrichum gloeosporioides* have been reported as causes of localized subcutaneous infections and artritis (Guarro *et al.,* 1999; Castro *et al.,* 2001; O’Quinn *et al.,* 2001; Kaliamurthy *et al.,* 2004; Cho *et al*., 2015); an unidentified *Colletotrichum *species as the cause of disseminated infection in a neutropenic patient (Midha *et al.,* 1996; Kaliamurthy *et al.,* 2004 ); and *Colletotrichum dematium*,* C. gloeosporioides*,* C. truncatum* and *Colletotrichum* sp. as causative agents of keratomycosis, most of them in India and Japan (Fernandez *et al.,* 2002; Shiraishi *et al.,* 2011; Squissato *et al.,* 2015).

Infections due to *C. dematium* and *C. gloeosporioides* respond clinically well to topical natamycin although previous reports suggested *in vitro* resistance to this antifungal, and to 5-flucytosine and fluconazole (Fernandez *et al.,* 2002; Kaliamurthy *et al.,* 2004; Mitani *et al.,* 2009; Shiraishi *et al.,* 2011). In contrast, voriconazole has been shown to have low MIC values but has not been effective in clinical use (Giaconi *et al.,* 2006). Combination therapy of voriconazole and natamycin may be an alternative treatment for keratitis due to *C. truncatum* (Al-Hatmi *et al*., 2016b).

To the best of our knowledge this is the first report of keratitis due to *C. truncatum* in Cuba and Latin-America and underlines the expanding spectrum of fungal agents causing eye infections.

**Table 1.** MIC values of eight antifungal agents versus* Colletotrichum* *truncatum* (CBS 134303 and CBS 134304) MIC values determined by the broth microdilution method according to M38-A2 guideline of the CLSI [Clinical and Laboratory Standards Institute (CLSI), 2008]. AMB, amphotericin B; FLU, fluconazole; ITR, itraconazole; VOR, voriconazole; POS, posaconazole; ISA, isavuconazole; ANI, anidulafungin; MICA, micafungin.

Isolates (CBS accession number)	MIC value (mg l^−1^) for antifungal agent/L							
	AMB	FLU	ITR	VOR	POS	ISA	ANI	MICA
134303	0.125	>64	>16	>16	4	4	4	2
134304	0.25	>64	16	8	2	2	4	1
